# Influence of Gluten-Free Diet on Gut Microbiota Composition in Patients with Coeliac Disease: A Systematic Review

**DOI:** 10.3390/nu14102083

**Published:** 2022-05-16

**Authors:** Iwona Kaliciak, Konstanty Drogowski, Aleksandra Garczyk, Stanisław Kopeć, Paulina Horwat, Paweł Bogdański, Marta Stelmach-Mardas, Marcin Mardas

**Affiliations:** 1 Department of Treatment of Obesity, Metabolic Disorders and Clinical Dietetics, Poznan University of Medical Sciences, 60-569 Poznan, Poland; ikaliciak@o2.pl (I.K.); drogowskos@wp.pl (K.D.); garczykaleksandra@gmail.com (A.G.); s.kopec@macron.pl (S.K.); paulina.horwat9@gmail.com (P.H.); pbogdanski@ump.edu.pl (P.B.); 2 Department of Gynecological Oncology, Institute of Oncology, Poznan University of Medical Sciences, 60-569 Poznan, Poland; marcin.mardas@ump.edu.pl

**Keywords:** coeliac disease, gluten-free diet, microbiome

## Abstract

The aim of this study was to assess the changes in microbiota composition during a gluten-free diet (GFD) in coeliac disease (CD) patients. The systematic search followed databases such as PUBMED (MEDLINE), SCOPUS, WEB OF SCIENCE and EMBASE. Out of 843 initially screened papers, a total number of 13 research papers were included. A total of 212 patients with CD on GFD, in comparison to 174 healthy individuals and 176 untreated patients with CD, were examined. Analysis of the microbial community based primarily on faecal samples and duodenal biopsies. *Bifidobacterium* was noticed to be less abundant in the study group than in both control groups, while the abundance of *Bacteroides* was more numerous in the group of CD patients on GFD. *Staphylococcaceae* prevailed in untreated CD patients. Despite the fact that the GFD was not able to fully restore commensal microorganism abundance, the treatment was associated with the greater abundance of selected beneficial bacteria and lower presence of pathogenic bacteria associated with worsening of CD symptoms.

## 1. Introduction

Coeliac disease (CD), described as a chronic autoimmune gluten intolerance, is becoming an increasingly important health problem for modern medicine in developed countries [1,2]. The occurrence of CD is strongly related to genetic factors, among which HLA class II plays a major role—HLA-DQ2 heterodimers are expressed in over 90% of patients, the remnant express HLA-DQ8 [3]. Nowadays, diagnosis of CD is primarily based on anti-tissue transglutaminase antibodies (tTG-IgA) testing and duodenal biopsy [4]. The symptom manifestations in patients suffering from CD especially concern the gastrointestinal tract, therefore differentiated dietary strategies are tested in the clinical setting i.e., for patients’ quality of life (QoL) improvement. Microbiological approaches have been considered as able to modulate the gluten-specific immune response. Furthermore, different biotechnological approaches based on the use of chemically/enzymatically modified gluten molecules have been proven effective in different models of CD.

A gluten-free diet (GFD) seems to be an effective way of CD treatment and enables the majority of patients to achieve clinical and histological remission [5]. According to recent data [6], children’s populations with CD are able to reconstruct up to 95% of their intestinal architecture within two years by following a GFD. However, some data suggest that treatment response in the adult population (30–60 years old) is less effective [7]. Among positive aspects of GFD application can be listed: positive influence on bone mineral density, increased weight-for-age z scores in the paediatric population diagnosed with both CD and type 1 diabetes and lower risk of depression in the female population [8,9,10]. A recent study suggested that the disturbance of the intestinal microbiota might be involved in the pathogenesis of CD. Altered microbiota might have an impact on immune response to gluten, with high release of proinflammatory cytokines [11]. Observed changes in intestinal microbiota composition that occur during the application of a GFD provide promising support for better QoL and outcomes in CD patients [12].

The aim of this study was to assess the changes in microbiota composition during a GFD in CD patients.

## 2. Experiment

### 2.1. Search Strategy, Inclusion and Exclusion Criteria

From August 2021 to December 2021, the literature of the following databases: PUBMED (MEDLINE), SCOPUS, WEB OF SCIENCE and EMBASE were searched in order to identify the interventional and observational studies that investigate differences in the gut microbiome in patients suffering from CD during the GFD application.

The search strategy was limited to the human population and English language. Original articles were included. No restrictions regarding the date of the publication or age of patients were used. Articles with low quality data or incomplete data that could not be fully obtained from authors were excluded.

The search strategy included the following index terms: *#1 Diet, Gluten Free OR Gluten-Free Diet OR Diets, Gluten-Free OR Gluten Free Diet OR Gluten-Free Diets; #2 Gastrointestinal Microbiomes OR Microbiome, Gastrointestinal OR Gut Microbiome OR Gut Microbiomes OR Microbiome, Gut OR Gut Microflora OR Microflora, Gut OR Gut Microbiota OR Gut Microbiotas OR Microbiota, Gut OR Gastrointestinal Flora OR Flora, Gastrointestinal OR Gut Flora OR Flora, Gut OR Gastrointestinal Microbiota OR Gastrointestinal Microbiotas OR Microbiota, Gastrointestinal OR Gastrointestinal Microbial Community OR Gastrointestinal Microbial Communities OR Microbial Community, Gastrointestinal OR Gastrointestinal Microflora OR Microflora, Gastrointestinal OR Gastric Microbiome OR Gastric Microbiomes OR Microbiome, Gastric OR Intestinal Microbiome OR Intestinal Microbiomes OR Microbiome, Intestinal OR Intestinal Microbiota OR Intestinal Microbiotas OR Microbiota, Intestinal OR Intestinal Microflora OR Microflora, Intestinal OR Intestinal Flora OR Flora, Intestinal OR Enteric Bacteria OR Bacteria, Enteric; #3 Disease, Celiac OR Gluten Enteropathy OR Enteropathies, Gluten OR Enteropathy, Gluten OR Gluten Enteropathies OR Gluten-Sensitive Enteropathy OR Enteropathies, Gluten-Sensitive OR Enteropathy, Gluten-Sensitive OR Gluten Sensitive Enteropathy OR Gluten-Sensitive Enteropathies OR Sprue, Celiac OR Sprue, Nontropical OR Nontropical Sprue OR Celiac Sprue OR Sprue.*

#1 AND #2 AND #3.

### 2.2. Data Extraction and Analysis

Initial revision to the titles of the articles was made by four researchers. Each researcher was responsible for searching one database. In the further stage abstracts were analysed and assessed for eligibility. Subsequently, the decision on the article inclusion was made collaboratively by all groups after the full text review.

From each qualified study the following data were extracted: a title, a main author, a publication year, a study name and design, countries involved, a total number of patients, age, sex, time of GFD treatment, antibiotic treatment, duration of the disease, presence of antigliadin and anti-transglutaminase antibodies (tGA) in serum and haplotypes within the HLA-DQ serotyping system. In order to examine the microbiome structure following methods were used: 16S rDNA sequencing, 16S rRNA sequencing, microscopic analysis and identification of *Bifidobacteria* by determination of fructose-6-phosphate phosphoketolase, Fluorescence in situ hybridization (FISH), flow cytometry, Fuzzy c-means (FCM), quantitative real-time polymerase chain reaction (qPCR), Short Chain Fatty Acid Analysis (SCFAs), Denaturing gradient gel electrophoresis (DGGE) and bacterial culture. Additionally, pH of stool samples was obtained.

Patients were assigned to one out of three groups according to health condition and diet: individuals affected by CD and following a GFD (CD on GFD); healthy individuals without CD and other known food intolerance who did not follow any particular diet (healthy control group, HC); individuals affected by CD who did not exclude gluten from the diet (untreated, UCD).

## 3. Results

### 3.1. Search Results

**Figure 1:**
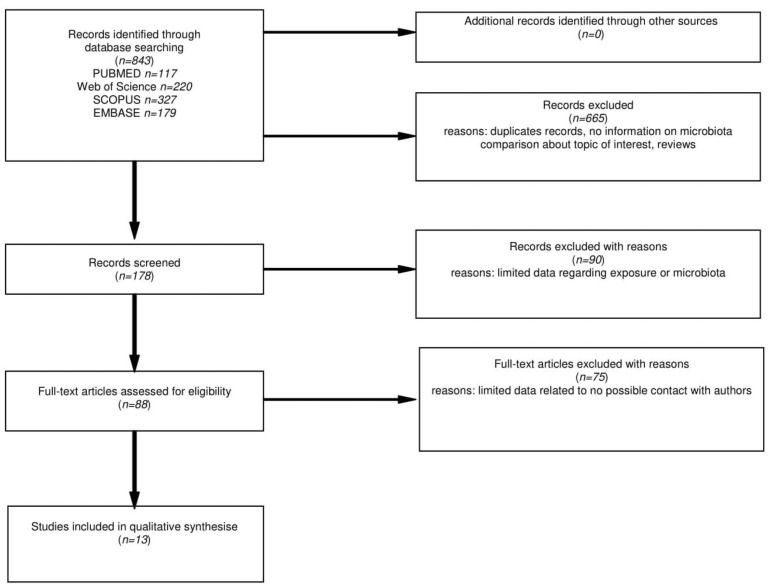
Flow chart of the databases search on microbiota changes in CD patients on GFD.

The flow chart for the literature search is presented in Figure 1. After the title database search, 843 articles were found, where further 178 abstracts were carefully examined. Finally, 88 full-texts were assessed and where needed, an attempt to contact the authors of papers with incomplete information was made. The detailed analysis of selected positions led to the acquisition of 13 papers that met all the criteria.

### 3.2. Characteristics of the Included Studies and Study Population

All papers were original intervention studies (Table 1). Most research was conducted on the European population [13,14,15,16,17,18,19,20,21,22,23]; however, patients from North America [24] and South America [25] were also included. Finally, gut microbiota of 492 patients with diagnosed CD and 299 healthy individuals was analysed. CD diagnosis was based on the presence of CD-specific antibodies and duodenal biopsy examination. Time of intervention with a GFD in individual studies varied widely: between 6–36 months based on the consumption of certified gluten-free food only [15].

The detailed clinical characteristic of studied patients was included in Table 2. The group of examined patients included both children [14,16,17,18,22,23,25] and adults [15,18,20,21]. The proportion of men and women in the eligible studies was comparable (50:50). In nine papers, antibiotic treatments were not allowed at least 1 month before collection of stool or/and duodenal biopsy samples [14,15,16,17,19,20,21,22,23,25]. Clinical symptoms of active CD such as bloating, abdominal pain, diarrhoea and weight loss were observed in untreated individuals in only two studies [22,23]. An anti-gliadin antibodies (AGA) test was positive in patients on a GFD in two [14,16] out of five studies, while in the untreated CD group, the AGA test was positive in two studies [22,23] reporting its presence. tGA test was positive in 25% of the research in which the test was carried out [14,16], whereas in the untreated CD group this ratio reached 100% of individuals [18,20,23]. Taking into account available data relating to haplotypes within the HLA-DQ serotyping system, more than 50% of patients affected by CD were DQ2+ or/and DQ8+ [21,22,23], while in all studies, less than 35% of the healthy control group was DQ2+ or/and DQ8+ [20,21]. Iron deficiency, which is one of the non-classical CD symptoms [15], was featured in only two papers [22,23], in an untreated CD group of patients.

### 3.3. Assessment of Microbiota Changes Related to GFD Treatment

Analysis of the microbial community was based on faecal samples and duodenal biopsies [13,14,15,16,17,18,19,20,21,22,23,24,25]. Two studies also used saliva [21] and blood [24] samples as biological material. The main route utilised by commensal bacteria to migrate to the bloodstream is through a damaged, inflamed, and therefore, permeable epithelium. It has been suggested that active CD patients, characterised by increased intestinal permeability, could acquire a unique blood microbiome reflecting the intestinal damage and that this phenomenon could influence their response to gluten. The differentiated microbiota composition with possible changes in three groups of individuals (CD on GFD, healthy controls and UCD) are presented in Table 3. Because of lack of taxonomic uniformity in studies included in the current research, a comparison of microbial communities between three groups of patients on one, we failed to obtain a common taxonomic rank. In general, *Bifidobacterium* genus, was the most frequently examined of all groups of bacteria [12,13,14,16,17,18,19,20,21,22,26,27] and occurred significantly less abundant in the study group than in healthy individuals according to faecal samples [13,19,20,22]. The same genus isolated from duodenal biopsy showed greater abundance in the healthy group than in the UCD [22]. Separate analysis on children and adult populations indicated that *Bifidobacterium* occurred less abundant in CD children in the GFD group and UCD group than in the healthy controls [13,17,22]. However, a similar tendency in the adult population was not observed. Studies on *Lactobacillus* changes [22,25] indicated significantly greater abundance in the GFD group than the HC [22,25]. Additionally, *Lactobacillus Sakei* was less abundant in CD for the GFD group than in both UCD and HC groups in the children’s population. The *Staphylococcus* genus isolated from duodenal biopsy was more abundant in UCD than CD for the GFD group and healthy group in only one study [22]. The same pattern of changes in faecal samples has been observed. Significant changes in abundance of *Bacteroides* genus assessed in faecal samples of children was found to be more pronounced in CD patients on a GFD than in healthy individuals [13,19,22]. In the case of duodenal biopsy, material tendency was observed in two out of three studies [13,22]. Significant changes in *E. coli* abundance was observed in three studies based on children’s populations [14,16,22], where two out of three studies showed domination of *E. coli* species in CD on the GFD group over remaining groups of patients [14,16,22]. After analysis of changes in *Firmicutes* phylum, no clear conclusions can be drawn. Two studies with faecal samples as biological material present opposite tendencies—domination of *Firmicutes* in the UCD group [21] and domination of *Firmicutes* in other groups over UCD group [24]. One study attached duodenal samples and a domination of *Firmicutes* in the HC over the CD for the GFD group was observed [15]. Data on *Bacteroidetes* phylum provided by Panelli et al. [21] showed larger abundance of *Bacteroidetes* in the CD for the GFD group than in the UCD and HC groups in both duodenal and faecal samples. Four studies reported statistically significant changes in the *Clostridium* group [17,19,21,22]; however, each of them investigated changes on different taxonomic ranks. Greater abundance of *Lactobacillus* and *Bacteroides* in the study group was found to be beneficial and presumably correlated with clinical remission [28]. *Bifidobacterium* genus was also proved to be beneficial in CD [29,30,31]; however, it was less abundant in comparison to the UCD and healthy controls in this study group.

## 4. Discussion

The impact of a GFD on the gut microbiome composition in CD patients has been proven by the obtained data. The approximation of the microbiome of CD patients to the composition of the microbiome of healthy people after excluding gluten from the diet has been found. However, the unequivocal trend of change and the resulting effects are difficult to define due to limited data.

Although it has not been thoroughly examined whether the transformation in human microbiome is either the cause or effect of CD, it certainly has an impact on inappropriate functioning of bowels and is related to the severity of clinical symptoms [29]. Microorganisms play a major role in the fermentation of indigestible food components into absorbable metabolites, the synthesis of essential vitamins, the removal of toxic compounds, the out competition of pathogens, the strengthening of the intestinal barrier and the stimulation and regulation of the immune system [32]. Gut microbiome structure more similar to healthy gut microbiome were observed in patients treated with a GFD in comparison to patients consuming gluten [14,17,19,23,24]. Distinctive mechanisms for immunomodulation by commensal microorganisms have been confirmed in the latest research. Short chain fatty acids (SCFA) that are produced by microbiota’s components affect Treg cells [33]. The abnormal butyrate production by microbiome is recognised as a cause of higher expression of non-functional form of FOXP3, which is associated with an enlarged risk of autoimmunity [34]. Disorders in T cell functions are successively underlined in the pathogenesis of CD [35,36]. T cell related production of antibodies against gluten peptides is an immune factor causing symptoms in CD. Mainly recognised antibodies that also have a main role in diagnosis are AGA and tGA. This result highlights that a reduction in gluten intake not only can alleviate clinical symptoms but can also impact the pathomechanism of disease.

The association between the microbiome composition in adult coeliac patients and the severity of clinical symptoms was demonstrated in study by Wacklin et al. [28]. *Firmicutes* and *Bacteroides* occurred to be significantly more abundant in the microbiome of asymptomatic patients, while *Proteobacteria*, *Acinetobacter* and *Neisseria* were more common among patients with gastrointestinal (GI) symptoms. Polled data on GFD indicated higher richness of *Bacteroides* [13,14,19,21,22] and *Firmicutes* [24] in treated patients’ samples compared to untreated individuals with CD. Furthermore, *Neisseria* [21] and *Proteobacteria* [24], which seems to be correlated with more severe symptoms, were less abundant among patients on GFD. The therapy’s potential to alleviate clinical symptoms of CD is presented by these facts.

*Bifidobacterium, Lactobacillus* and *Bacteroides* play out a significant role in being a part of intestinal microbiota. The *Bifidobacterium* genus, which belongs to Acinetobacter phylum, takes part in acetate synthesis and prevents *E. coli* colonisation as a commensal [29]. *Bifidobacterium* seems to be very beneficial in autoimmune GI diseases such as CD. *Bifidobacterium* strains have abilities to neutralise the toxicity of gliadin and alleviate mechanical damages in gut walls triggered by gluten [30]. Sjogren et al. [31] assessed that high abundance of *Bifidobacterium* species in the faecal samples corresponds significantly with the IgA level in the saliva of examined infants [31], which has a direct impact on increased protection against allergies and autoimmunity [37]. Nonetheless, the *Bifidobacterium* genus was less abundant in the study group of CD patients compared to the control groups regardless of the type of sample [13,19,20,21,22]. This fact may reveal that even long-term and strict adherence to the GFD may not be sufficient to completely restore the microbiome composition to show the similarity with a related control population. This result is partially consistent with the outcomes obtained by Sanz Y. et al. [38]. The *Bacteroides* genus turned out to be more abundant in the treated CD group than control groups [13,19,21,22], which seems to be beneficial as it is associated with host protection against pathogenic microbes and the delivery of nutrients for commensal microflora [39]. *Lactobacillus* bacteria are involved in many various functions in the human intestine, such as antibiotic production, organic acid production, bile deconjugation and carcinogen suppression [40]. Additionally, it was reported that *Lactobacillus crispatus* confers an anti-inflammatory phenotype to human dendritic cells, which is especially profitable in inflammatory diseases such as CD [41]. A statistically significant higher abundance of *Lactobacillus* in the study group than in the UCD group was described in two papers [22,25]. Furthermore, the proportion of *Lactobacillus* species plays an important role and its disturbance may go undetected with the preserved abundance [38]. Finally, time of intervention on a GFD might play an important role, which was already suggested by Garcia-Mazcorro JF et al. [42]. There were no significant differences between GI microbiome composition before and after GFD treatment over 4 weeks in a dietary intervention. Nevertheless, studies included in the current research can be characterised as long term if an intervention lasted at least 6 months. Unfortunately, detailed information on patients’ diets or on methods of controlling dietary compliance was provided by the authors. Only Panelli et al. [21] included information about using a five-level score to evaluate patients’ adherence to GFD. However, this scale is a subjective tool, while it is comprised of a dietary questionnaire. This is a serious obstacle to the conduct of reliable research, because strict adherence to GFD is required to obtain potential beneficial effects. Furthermore, difficulties with controlling a GFD in many CD patients are highlighted by researchers [43], which indicates the need for strict and objective control.

## 5. Limitations

This study has distinct limitations. Firstly, all papers included were cross-sectional. The impact on gut microbiome could evolve depending on time that elapsed since the implementation of the GFD, which requires a special caution during interpretation. Additionally, deficient data about clinical symptoms as well as serological and genetic profile did not provide a complete view of the patients. Another limitation was that there was no possibility to fully control the patients’ diets. Even though coeliac patients are well educated about GFD, there is never full assurance whether they consume (intentionally or not) even the minimum doses of gluten. Moreover, a lack of information about required time without any antibiotic interventions before being examined [13,15,18,24] or the short duration of this period [16,17,19,20,21,22,23,25] may raise doubts, as it is known that antibiotics have a significant and long-term impact on gut microbiome composition and functions [44]. Restrictions regarding the consumption of probiotics by the examined patients were introduced only in three studies [13,19,21]. Probiotics affect the composition and functioning of the microbiome in many ways [45], thus its intake should be controlled more strictly. In general, the diet quality was not evaluated in the selected studies, although it may directly influence the gut microbiota composition. Finally, different sequencing methods used in the single studies may have yielded different results and this may have impacted the overall analysis.

## 6. Conclusions

In conclusion, patients suffering from CD who follow a GFD correlates with the presence of a gut microbiome composition similar to healthy individuals. However, full restoration of commensal microorganism abundance in patients treated with GFD was not observed.

## Figures and Tables

**Table 1:** Characteristics of the included studies (*n* = 13).

Study	Year	Country	Study Design	CD on GFD	Healthy	UCD	Duration of Treatment with Gluten-Free Diet (Months)
Nadal I. et al. [16]	2007	Spain	CSS	10	8	20	12–24
Collado M. et al. [22]	2008	Spain	CSS	18	30	30	min 24
Di Cagno R. et al. [19]	2009	Italy	CSS	7	7	7	min 24
Schippa S. et al. [14]	2010	Italy	CSS	20	10	20	9
De Palma G. et al. [17]	2010	Spain	CSS	18	20	24	min 24
Di Cagno R. et al. [13]	2011	Italy	CSS	19	15	0	min 24
Kalliomäki M. et al. [18]	2012	Finland	CSS	6	9	10	min 12
Nistal E. et al. [20]	2012	Spain	CSS	11	11	10	min 24
Sanchez E. et al. [23]	2013	Spain	CSS	17	8	32	min 24
Pirjo W. et al. [15]	2014	Finland	CSS	34	0	0	min 36
Lorenzo Pisarello M.J. et al. [25]	2015	Argentina	CSS	15	15	0	min 6
Serena G. et al. [24]	2019	USA	CSS	8	10	10	min 6
Panelli S. et al. [21]	2020	Italy	CSS	29	31	13	36 (median)

CD—coeliac disease, GFD—gluten-free diet, CSS—cross-sectional study, CD on GFD—patients with CD on GFD, Healthy—healthy individuals without CD and other known food intolerance, UCD—untreated CD patients.

**Table 2:** Characteristics of the study population (*n* = 212).

Study	Age (Years) Mean ±SD			Sex (% Male)			Antibiotic Treatment	AGA			tGA			HLA		
	CD on GFD	Healthy	UCD	CD on GFD	Healthy	UCD		CD on GFD	Healthy	UCD	CD on GFD	Healthy	UCD	CD on GFD	Healthy	UCD
Di Cagno R. et al. [13]	9.7 (6–12) ^1^	10.4 (6–12) ^1^	N/A	42	47	N/A	no last 3 mo	N/A	N/A	N/A	N/A	N/A	N/A	N/A	N/A	N/A
Lorenzo Pisarello M.J. et al. [25]	7.5	6.5	N/A	N/A	N/A	N/A	no last 1 mo	negative	N/A	N/A	negative	N/A	N/A	N/A	N/A	N/A
Schippa S. et al. [14]	N/A	11.7 (7.8–20.8)	8.3 (1.2–16.1)	40	30	40	no last 3 mo	positive	N/A	N/A	positive	N/A	N/A	N/A	N/A	N/A
Pirjo W. et al. [15]	N/A	N/A	N/A	N/A	N/A	N/A	No	N/A	N/A	N/A	0.3	0.8	N/A	65% DQ2/DQ8	N/A	N/A
Nadal I. et al. [16]	5.1	4.1	N/A	N/A	N/A	N/A	no last 1 mo	positive	N/A	N/A	positive	N/A	N/A	N/A	N/A	N/A
De Palma G. et al. [17]	5.5	5.3	5.5	N/A	N/A	N/A	no last 1 mo	N/A	N/A	N/A	N/A	N/A	N/A	N/A	N/A	N/A
Kalliomäki M. et al. [18]	46	8.5	9.5	N/A	N/A	N/A	N/A	N/A	N/A	N/A	negative	negative	positive	N/A	N/A	N/A
Di Cagno R. et al. [19]	N/A	N/A	N/A	N/A	N/A	N/A	no last 1 mo	N/A	N/A	N/A	N/A	N/A	N/A	N/A	N/A	N/A
Nistal E. et al. [20]	40.4	30.9	38.5	N/A	N/A	N/A	no last 1 mo	N/A	N/A	N/A	negative	negative	positive	N/A	0% DQ2/DQ8	N/A
Panelli S. et al. [21]	37+/−6	44+/−17	35+/−6	31	23	15	no last 1 mo	N/A	N/A	N/A	N/A	N/A	N/A	58% DQ2+, 3% DQ8+, 7% DQ2/DQ8+	32% DQ2+, 10% DQ2+, 3% DQ2/DQ8+, 26% DQ2/DQ8-	62% DQ2+, 0% DQ8+, 23% DQ2/DQ8+
Serena G. et al. [24]	N/A	N/A	N/A	N/A	N/A	N/A	no last 1 mo	N/A	N/A	N/A	negative	N/A	positive	N/A	N/A	N/A
Collado M. et al. [22]	5.43	3.75	4.7	44.4	43.3	40	no last 1 mo	negative	negative	positive	negative	negative	positive	DQ2+-100%	N/A	DQ2+-100%
Sanchez E. et al. [23]	5.9	6.9	5.1	47.1	50	43.7	no last 1 mo	negative	negative	positive	negative	negative	positive	DQ2/DQ8 100%	N/A	DQ2/DQ8 100%

N/A—not applicable, AGA—anti-gliadin antibodies, tGA—anti-tissue transglutaminase antibodies, HLA—haplotypes within the HLA-DQ serotyping system (genetic test), CD—coeliac disease, GFD—gluten-free diet, CD on GFD—patients with CD on GFD, Healthy—healthy individuals without CD and other known food intolerance, UCD—untreated CD patients. ^1^ Median age (range).

**Table 3:** Gut microbiota diversity based on GFD application in CD patients.

Study	Type of Samples	Method	Abundance			*p*-Value
			CD on GFD	Healthy	UCD	
Di Cagno R. et al. [13]	Duodenal biopsy and faecal samples	16S rRNA sequencing	*Bifidobacteria* 5.34	*Bifidobacteria* 6.72	N/A	*p* = 0.023
			*Lactobacilli* 8.1	*Lactobacilli* 8.6		*p* > 0.05
			*Enterococci* 7.83	*Enterococci* 8.23		*p* > 0.05
			*Bacteroides* 6.02	*Bacteroides* 5		*p* = 0.014
			*Staphylococci* 7.6	*Staphylococci* 7		*p* > 0.05
			*Salmonella*, *Shigella*, *Klebsiella* 7.26	*Salmonella*, *Shigella*, *Klebsiella* 7.3		*p* > 0.05
			*Enterobacteria* 6.7	*Enterobacteria* 6.4		*p* > 0.05
			*Clostridium* 1 ^1^	*Clostridium* 1 ^1^		*p* > 0.05
Lorenzo Pisarello M.J. et al. [25]	Faecal samples	bacterial culture	*Anaerobic* (1.37 ± 5.47) × 109 CFU/g	*Anaerobic* (2.09 ± 9.08) × 109 CFU/g	N/A	*p* > 0.05
			*Aerobic* (1.54 ± 5.47) × 109	*Aerobic* (3.31 ± 2.57) × 109		*p* > 0.05
			*Enterobacteria* (1.18 ± 7.69) × 106 CFU/g	*Enterobacteria* (6.60 ± 5.23) × 105 CFU/g		*p* > 0.05
			*Lactobacillus* (4.38 ± 3.14) × 105	*Lactobacillus* (4.00 ± 2.45) × 106;		*p* < 0.05
Schippa S. et al. [14]	Duodenal biopsy	16S rDNA Sequencing	*Bacteroides vulgatus* 85%	*Bacteroides vulgatus* 94.7%	Bacteroides vulgatus 20%	*p* < 0.001
			*E. coli* 95%		*E. coli* 20%	*p* < 0.001
			*Bifidobacterium* 30%	Parabacteroides distasonis 0 ^6^	*Bifidobacterium* 20% ^6^	*p* > 0.05
			Parabacteroides distasonis 31.6% ^6^			*p* = 0.009
Pirjo W. et al. [15]	Duodenal biopsy	16S rDNA Sequencing	*Bacteroidetes* 15%	*Bacteroidetes* 25%	N/A	*p* = 0.01
			*Firmicutes* 33%	*Firmicutes* 46%		*p* = 0.05
			*Proteobacteria* 40%	*Proteobacteria* 21%		*p* = 0.04
			72 OTUs per sample	106 OTUs per sample		
Nadal I. et al. [16]	Duodenal biopsy	FISH and flow cytometry	Bacteroides–Prevotella 12.98% ^2^	Bacteroides–Prevotella 6.07	Bacteroides–Prevotella 4.52	*p* = 0.027
			*Escherichia coli* 10.98% ^2^	*Escherichia coli* 5.04	*Escherichia coli* 4.1	*p* = 0.027
			Streptococcus–Lactococcus 10.88% ^2^	Streptococcus–Lactococcus 7.18	Streptococcus–Lactoco 9.44	*p* > 0.05
			*Bifidobacterium* 9.24% ^2^	*Bifidobacterium* 10.55	*Bifidobacterium* 4.32	*p* > 0.05
De Palma G. et al. [17]	Faecal samples	FISH and FCM	*Bifidobacterium* 9.20% ^2^	*Bifidobacterium* 12.54% ^2^	*Bifidobacterium* 7.73% ^2^	*p* = 0.009
			*C. histolyticum* 9.41% ^2^	*C. histolyticum* 11.61% ^2^	*C. histolyticum* 5.26% ^2^	*p* = 0.031
			*C. lituseburense* 4.41% ^2^	*C. lituseburense* 6.83% ^2^	C. lituseburense 3.23% ^2^	*p* = 0.024
			*Lactobacillus-Enterococcus* 1.12% ^2^	*Lactobacillus-Enterococcus* 1.76% ^2^	*Lactobacillus-Enterococcus* 1.94% ^2^	*p* > 0.05
			*Staphylococcus* 16.49% ^2^	*Staphylococcus* 18.04% ^2^	*Staphylococcus* 10.36% ^2^	*p* > 0.05
			Bacteroides-Prevotella 2.61% ^2^	Bacteroides-Prevotella 2.32% ^2^	Bacteroides-Prevotella 3.54% ^2^	*p* = 0.033
			*E. coli* 6.39% ^2^	*E. coli* 7.32% ^2^	*E. coli* 5.2% ^2^	*p* > 0.05
			*F. prausnitzii* 11.09% ^2^	*F. prausnitzii* 13.88% ^2^	*F. prausnitzii* 6.03% ^2^	*p* = 0.045
			*Sulphate-reducing* bacteria 9.82% ^2^	*Sulphate-reducing* bacteria 10.02% ^2^	*Sulphate-reducing* bacteria 9.58% ^2^	*p* > 0.05
Kalliomäki M. et al. [18]	Small intestinal biopsy	qPCR	Bacteroides-Prevotella-Porphyromona group 1682	Bacteroides-Prevotella-Porphyromonas group 684	Bacteroides-Prevotella-Porphyromonas group 834	*p* > 0.05
			*Bifidobacterium* genus 140	*Bifidobacterium* genus 190	*Bifidobacterium* genus 234	*p* > 0.05
			*Bifidobacterium adolescentis* 5 ^5^	*Bifidobacterium adolescentis* 14 ^5^	*Bifidobacterium adolescentis* 10 ^5^	*p* > 0.05
Di Cagno R. et al. [19]	Faecal samples	16S rRNA sequencing RAPD-PCR analysis	*Lactic acid* bacteria 8.09	*Lactic acid* bacteria 8.89	*Lactic acid* bacteria 8.02	*p* > 0.05
			*Bifidobacterium* 6.83	*Bifidobacterium* 7.88	*Bifidobacterium* 5.51	*p* = 0.03
			*Bacteroides* 8.31	*Bacteroides* 7.05	*Bacteroides* 8.69	*p* = 0.045
			*Clostridium* 8.07	*Clostridium* 5.50	*Clostridium* 8.04	*p* = 0.045
			Staphylococcus/Micrococcus 7.42	Staphylococcus/Micrococcus 8.05	Staphylococcus/Micrococcus 6.00	*p* > 0.05
			*Enterobacteriaceae*	*Enterobacteriaceae* 8.05	*Enterobacteriaceae* 6.69	*p* > 0.05
			Total anaerobes 9.63 ^1^	Total anaerobes 10.03 ^1^	Total anaerobes 9.87 ^1^	*p* > 0.05
Nistal E. et al. [20]	Faecal samples	SCFAs, DGGE	*Lactobacillus sakei* 0%	*Lactobacillus sakei* 45%	*Lactobacillus sakei* 40%	*p* < 0.05
			*Bifidobacterium bifidum* 18%	*Bifidobacterium bifidum* 9%	*Bifidobacterium bifidum* 60%	*p* < 0.05
			*Bifidobacterium catenulatum* 18%	*Bifidobacterium catenulatum* 36%	*Bifidobacterium catenulatum* 80%	*p* < 0.05
			*Bifidobacterium* sp.0% ^6^	*Bifidobacterium* sp. 45% ^6^	*Bifidobacterium* sp. 20% ^6^	*p* < 0.05
Panelli S. et al. [21]	Saliva samples, duodenal biopsy and faecal samples	16S rRNA sequencing	Duodenal samples:	duodenal samples	duodenal samples:	
			*Bacteroidetes* 28.08%	*Bacteroidetes* 20.76%	*Bacteroidetes* 18.20%	*p* < 0.05
			*Actinobacteria* 7.94%	*Actinobacteria* 11.1%	*Actinobacteria* 4.15%	*p* < 0.05
			*Proteobacteria* 19.21%	*Proteobacteria* 17.89%	*Proteobacteria* 35.48%	*p* < 0.05
			*Streptococcaceae* 18.34%	*Streptococcaceae* 25.77%	*Streptococcaceae* 22.77%	*p* < 0.05
			*Gemellaceae* 1.51%	*Gemellaceae* 2.17%	*Gemellaceae* 0.83%	*p* < 0.05
			*Veillonellaceae* 8.95%	*Veillonellaceae* 7.37%	*Veillonellaceae* 4.50%	*p* < 0.05
			*Lachnospiraceae* 3.26%	*Lachnospiraceae* 2.71%	*Lachnospiraceae* 2.00%	*p* < 0.05
			*Prevotellaceae* 17.8%	*Prevotellaceae* 12.1%	*Prevotellaceae* 6.80%	*p* < 0.05
			*Micrococcaceae* 4.98%	*Micrococcaceae* 7.51%	*Micrococcaceae* 2.27%	*p* < 0.05
			*Neisseriaceae* 7.91%	*Neisseriaceae* 3.95%	*Neisseriaceae* 16.14%	*p* < 0.05
			Stool samples:	Stool samples:	Stool samples:	
			*Bacteroidetes* 59.99%	*Bacteroidetes* 51.97%	*Bacteroidetes* 44.27%	*p* < 0.05
			*Firmicutes* 34.21%	*Firmicutes* 36.39%	*Firmicutes* 47.83%	*p* < 0.05
			*Actinobacteria* 0.82%	*Actinobacteria* 1.96%	*Actinobacteria* 2.93%	*p* < 0.05
			*Proteobacteria* 3.96%	*Proteobacteria* 6.9%	*Proteobacteria* 3.12%	
			*Coriobacteriaceae* 0.12%	*Coriobacteriaceae* 0.14%	*Coriobacteriaceae* 1.39%	*p* < 0.05
			*Clostridiaceae* 0.57%	*Clostridiaceae* 0.18%	*Clostridiaceae* 0.63%	*p* < 0.05
			*Veillonellaceae* 6.35%	*Veillonellaceae* 6.35%	*Veillonellaceae* 2.4%	*p* < 0.05
			*Erysipelitrichaceae* 0.44%	*Erysipelitrichaceae* 0.30%	*Erysipelitrichaceae* 1.14%	*p* < 0.05
			*Ruminococcaceae* 13.94%	*Ruminococcaceae* 23.52%	*Ruminococcaceae* 23.52%	*p* < 0.05
			*Coriobacteriaceae* 0.12%	*Coriobacteriaceae* 0.14%	*Coriobacteriaceae* 1.39%	*p* > 0.05
			*Enterobacteriaceae* 2.13%	*Enterobacteriaceae* 0.46%	*Enterobacteriaceae* 1.84%	*p* > 0.05
			*Pasteurellaceae* 0.41%	*Pasteurellaceae* 2.32%	*Pasteurellaceae* 0.56%	*p* > 0.05
Serena G. et al. [24]	Blood samples and faecal samples	16S rRNA sequencing	blood samples:	blood samples:	blood samples:	
			*Proteobacteria* 41.26%	*Proteobacteria* 42.34%	*Proteobacteria* 49.16%	*p* > 0.05
			*Actinobacteria* 8.16%	*Actinobacteria* 8.42%	*Actinobacteria* 9.44%	*p* > 0.05
			*Bacteroidetes* 6.39%	*Bacteroidetes* 5.87%	*Bacteroidetes* 7.87%	*p* > 0.05
			*Firmicutes* 36.36%	*Firmicutes* 32.07%	*Firmicutes* 26.44%	*p* > 0.05
			Other 7.83%	Other 11.3%	Other 7.09%	*p* > 0.05
			faecal samples:	faecal samples:	faecal samples:	
			*Firmicutes* 71.41%	*Firmicutes* 77.37%	*Firmicutes* 57.8%	*p* < 0.05
			*Bacteroidetes* 11.87%	*Bacteroidetes* 13.01%	*Bacteroidetes* 31.86%	*p* < 0.05
			Other 16.72%	Other 9.62%	Other 10.34%	*p* > 0.05
Collado M. et al. [22] ^3^	Duodenal biopsy and faecal samples	qPCR	Study—Treated CD faecal samples:	Healthy faecal samples:	Untreated CD faecal samples:	
			*Bifidobacterium*—8.77 (8.58–9.60)	*Bifidobacterium*—9.80 (9.23–10.33)	*Bifidobacterium*—8.67 (8.68–9.90)	*p* < 0.05
			*Bacteroides*—8.55 (8.30–8.90)	*Bacterioides*—8.13 (7.41–8.53)	*Bacterioides*—8.71 (8.05–9.00)	*p* < 0.05
			*Staphylococcus*—6.58 (6.28–6.88)	*Staphylococcus*—6.78 (6.26–7.18)	*Staphylococcus*—7.07 (6.06–7.35)	*p* < 0.05
			*C. coccoides*—9.00 (8.41–9.56)	*C. coccoides*—9.00 (8.23–9.79)	*C. coccoides*—9.03 (8.50–9.52)	*p* > 0.05
			*C. leptum*—9.17 (8.86–9.74)	*C. leptum*—8.42 (7.89–8.74)	*C. leptum*—8.88 (8.10–9.50)	*p* < 0.05
			*Lactobacillus*—6.68 (6.26–7.30)	*Lactobacillus*—6.39 (6.08–6.85)	*Lactobacillus*—6.34 (6.06–6.95)	*p* < 0.05
			*E. coli*—7.05 (6.20–7.64)	*E. coli*—6.40 (6.21–6.56)	*E. coli*—7.11 (6.50–8.01)	*p* < 0.05
			*A. muciniphilia*—7.01 (5.80–7.44)	*A. muciniphilia*—5.75 (4.96–7.40)	*A. muciniphilia*—7.00 (5.65–8.00)	*p* > 0.05
			duodenal biopsy:	duodenal biopsy:	duodenal biopsy:	
			*Bifidobacterium*—6.15 (4.97–6.28)	*Bifidobacterium*—6.27 (6.03–6.80)	*Bifidobacterium*—5.95 (5.55–6.21)	*p* < 0.05
			*Bacterioides*—4.98 (3.98–5.00)	*Bacteroides*—3.28 (2.25–4.10)	*Bacteroides*—4.97 (4.03–5.20)	*p* < 0.05
			*Staphylococcus*—2.67 (2.12–3.00)	*Staphylococcus*—2.35 (1.25–2.77)	*Staphylococcus*—3.97 (3.44–4.06)	*p* < 0.05
			*C. coccoides*—3.70 (3.30–4.12)	*C. coccoides*—4.06 (3.70- 4.70)	*C. coccoides*—4.00 (3.65–4.25)	*p* > 0.05
			*C. leptum*—3.98 (3.23–4.15)	*C. leptum*—3.65 (3.05–4.52)	*C. leptum*—4.56 (4.42–4.70)	*p* < 0.05
			*Lactobacillus*—2.70 (2.58–3.46)	*Lactobacillus*—3.12 (2.74–4.14)	*Lactobacillus*—4.92 (4.16–5.25)	*p* < 0.05
			*E. coli*—3.18	*E. coli*—3.04	*E. coli*—4.23 (3.99–4.47)	*p* < 0.05
			*A. muciniphila*—N/A	*A. muciniphila*—2.78 (2.50–3.38)	*A. muciniphila*—2.95 (2.74—4.00)	*p* > 0.05
Sanchez E. et al. [23] ^1^	Duodenal biopsy	16S rRNA sequencing	*Enterobacteriaceae*—4	*Enterobacteriaceae*—0	*Enterobacteriaceae*—22	N/A
			*Actinobacteria*—2	*Actinobacteria*—4	*Actinobacteria*—15	
			*Staphylococcaceae*—8	*Staphylococcaceae*—2	*Staphylococcaceae*—32	
			*Streptococcaceae*—58	*Streptococcaceae*—58	*Streptococcaceae*—59	
			*Clostridiaceae*—2	*Clostridiaceae*—3	*Clostridiaceae*—4	
			*Lactobacillaceae*—0	*Lactobacillaceae*—2	*Lactobacillaceae*—0	
			*Enterococcaceae*—0	*Enterococcaceae*—0	*Enterococcaceae*—2	
			*Veillonellaceae*—2	*Veillonellaceae*—0	*Veillonellaceae*—3	
			*Carnobacteriaceae*—4 ^4^	*Carnobacteriaceae*—1 ^4^	*Carnobacteriaceae*—2 ^4^	

^1^ Cultivable cells (log cfu/g) of the main microbial groups in faecal samples. ^2^ Median (%). ^3^ Data are shown as medians and IQR of log of cell numbers per gram of samples. ^4^ Abundance is expressed as the absolute numbers of isolated clones belonging to one specific taxonomic group. ^5^ Medians of 16S rRNA gene copies per milligram of tissue. ^6^ Percentage of patients with bacteria detected. N/A—not applicable, CD—coeliac disease, GFD—gluten-free diet, CD on GFD—patients with CD on GFD, Healthy—healthy individuals without CD and other known food intolerance, UCD—untreated CD patients, FISH—Fluorescence in situ hybridization, SCFAs—Short Chain Fatty Acid Analysis, DGGE—Denaturing gradient gel electrophoresis, FCM—Fuzzy c-means.

## Data Availability

Detailed secondary data will be available after direct contact with stelmach@ump.edu.pl.
